# Heat-Attributable Deaths between 1992 and 2009 in Seoul, South Korea

**DOI:** 10.1371/journal.pone.0118577

**Published:** 2015-02-18

**Authors:** Clara T. Kim, Youn-Hee Lim, Alistair Woodward, Ho Kim

**Affiliations:** 1 School of Public Health & Institute of Health and Environment, Seoul National University, Seoul, Republic of Korea; 2 Institute of Environmental Medicine, Seoul National University Medical Research Center & Environmental Health Center, Seoul National University College of Medicine, Seoul, Republic of Korea; 3 School of Population Health, University of Auckland, Auckland, New Zealand; Georgia State University, UNITED STATES

## Abstract

**Background:**

Climate change may significantly affect human health. The possible effects of high ambient temperature must be better understood, particularly in terms of certain diseases’ sensitivity to heat (as reflected in relative risks [RR]) and the consequent disease burden (number or fraction of cases attributable to high temperatures), in order to manage the threat.

**Purpose:**

This study investigated the number of deaths attributable to abnormally high ambient temperatures in Seoul, South Korea, for a wide range of diseases.

**Method:**

The relationship between mortality and daily maximum temperature using a generalized linear model was analyzed. The threshold temperature was defined as the 90^th^ percentile of maximum daily temperatures. Deaths were classified according to ICD-10 codes, and for each disease, the RR and attributable fractions were determined. Using these fractions, the total number of deaths attributable to daily maximum temperatures above the threshold value, from 1992 to 2009, was calculated. Data analyses were conducted in 2012–2013.

**Results:**

Heat-attributable deaths accounted for 3,177 of the 271,633 deaths from all causes. Neurological (RR 1.07; 95% CI, 1.04–1.11) and mental and behavioral disorders (RR 1.04; 95% CI, 1.01–1.07) had relatively high increases in the RR of mortality. The most heat-sensitive diseases (those with the highest RRs) were not the diseases that caused the largest number of deaths attributable to high temperatures.

**Conclusion:**

This study estimated RRs and deaths attributable to high ambient temperature for a wide variety of diseases. Prevention-related policies must account for both particular vulnerabilities (heat-sensitive diseases with high RRs) and the major causes of the heat mortality burden (common conditions less sensitive to high temperatures).

## Introduction

The progression of climate change has led to a concomitant worldwide increase in the incidence of extreme heat spells. Following several particularly dramatic examples, such as the heat waves in Chicago (1995) and Western Europe (2003), many studies have been published on high temperatures and human health.[1–4]

It has been well documented that exposure to elevated ambient temperatures significantly increases the risk of mortality from cardiovascular problems.[5–8] The normal human body temperature ranges from 36.1°C to 37.8°C and is maintained by the hypothalamus in the brain. When the degree of environmental heat exceeds the regulatory capacity of the hypothalamus, the body’s core temperature rises and exerts substantial stress on the organs, particularly the cardiovascular system.[6, 8–10] Most studies on the relationship between high temperature and mortality have focused on overall death and examined temperature-related cardiovascular and respiratory conditions, since these account for the majority of fatalities.[6, 7, 11–15] While some evidence suggests a substantial effect of high temperatures on mortality rate,[13, 14, 16–20] the relationship between heat and specific causes of death has not yet been examined in a metropolitan city in Asia.

In South Korea, an extreme heat watch/warning system was launched in 2007. These heat warning systems mainly concentrate on servicing the elderly, nursing homes, and childcare professionals. Present results show that heat affects a broad range of diseases and conditions, suggesting that there needs to be further development of inclusive heat-associated mortality prevention policies and programs for specific diseases.

Prior studies on temperature and health employed a variety of statistical designs and modeling, such as case-control,[1, 2, 9] time series,[6, 12, 21–23] and case-crossover[18, 21, 22, 24] methods. There has been extensive research on estimating threshold temperatures and applying relative risk (RR) derived from time series analysis.[11, 15, 23, 25–27] Of the many previous epidemiological analyses on high temperatures, few have used both relative risk and attributable death to evaluate the effect of high temperature on mortality according to disease state.[18, 19, 28–31]“Attributable death” refers to the number of deaths that would be averted if a given risk factor was absent. [32] Estimating the number of deaths attributable to high temperatures for specific diseases will enable policymakers to anticipate the health gains that can be achieved from the implementation of preventative measures. [4, 33, 34]

Unlike previous studies that focused intensively on identifying the RRs of highly prevalent diseases, the present study investigates not just the RRs, but a number of heat-attributable deaths that occurred in conjunction with cause-specific disease in Seoul, South Korea. Quantifying both RRs and attributable deaths will provide an understanding of the high temperature effect and the burden of mortality attributable to heat.

## Methods

### Study Area

Seoul is the largest city in South Korea, with a reported population of 9,794,304 in 2010 (S1 Fig.). The total land area of the South Korea is 99,720 square kilometers. The territories of Seoul are about 605.17 square kilometers. It is the same size as the city and county of San Francisco. The population density of Seoul is 17,473 person per square kilometers in 2010. It is more than 2.5 times the population density of San Francisco (6,664 person per square kilometer). [35] Seoul has a humid, subtropical climate and four distinct seasons. The East Asian monsoon affects the weather throughout the year,[36] approaching from the southeast region of the Korean Peninsula during the summer (June to August) and bringing hot and humid weather with abundant precipitation (accounting for more than 60% of annual rainfall).[36, 37] The “urban heat island effect” is particularly marked in Seoul because the main commercial and industrial sectors are located there.[38] Over the past century, the average annual temperature has risen by 1.7°C in South Korea, greater than the increase in global average temperatures (0.74°C).[39] In Korea, heat wave advisories have been issuing warnings from summer 2007. The warning is issued if the temperature is expected to remain above 35 degrees Celsius for two or more consecutive days. The Korean climate change assessment reported that consecutive days of very high temperatures are increasing in magnitude and duration.[40]

### Mortality Data

This study examined the influence of temperature on mortality by disease category. The daily mortality data from 1992 to 2009 were provided by Statistics Korea[35] (Table 1). Deaths were classified in accordance with International Classification of Disease, 10^th^ Revision (ICD-10) codes (Table 1).

**Table 1:** The total and daily average mortality from 1992 to 2009 in Seoul.

Main-category Diseases	Subcategory Diseases	ICD-10	All-season			Warm Season^a^		
			Death (Count)	Daily Mean(SD)	Range^b^	Death (Count)	Daily Mean(SD)	Range
All cause			676,509	102.91(12.58)	61–174	271,633	98.67(11.86)	61–174
External causes of morbidity and mortality and injury(Accidental causes)		S,T,V01-Y98	80,033	12.17(4.32)	1–81	35,557	12.92(4.49)	2–79
	Transport accidents	V01-V99	22,220	3.38(2.53)	0–65	9,769	3.55(2.76)	0–65
All cardiovascular		I00-I99	169,882	25.84(6.07)	8–59	65,675	23.85(5.65)	8–59
	Ischemic heart disease	I20-I25	32,257	4.91(2.55)	0–16	12,661	4.6(2.4)	0–15
	Hypertensive diseases	I10-I15	11,413	1.74(1.41)	0–9	4,371	1.59(1.35)	0–9
	Heart failure	I50	6,758	1.03(1.19)	0–9	2,797	1.02(1.19)	0–9
	Myocardial Infarction	I20-I23	27,288	4.15(2.28)	0–15	10,664	3.87(2.14)	0–12
	Stroke, Cerebrovascular diseases	I60-I69	96,947	14.74(4.53)	3–35	37,286	13.54(4.27)	3–35
	Chronic ischemic heart disease	I05-I09	4,871	0.74(0.92)	0–7	1,962	0.71(0.89)	0–6
	Sudden Death	I46	8,678	1.32(1.63)	0–15	3,178	1.15(1.41)	0–11
Respiratory System		J00-J99	34,904	5.31(2.53)	0–21	13,440	4.88(2.28)	0–14
	Asthma	J45-J46	6,748	1.03(1.05)	0–7	2,570	0.93(0.98)	0–6
	COPD	J40-J44	10,566	1.61(1.3)	0–9	4,067	1.48(1.22)	0–7
	Pneumonia	J09-J22	10,114	1.54(1.37)	0–11	3,876	1.41(1.3)	0–9
Endocrine, nutritional and metabolic diseases		E00-E99	30,685	4.67(2.35)	0–17	11,412	4.14(2.15)	0–15
	Diabetes mellitus	E10-E14	28,123	4.28(2.27)	0–16	10,375	3.77(2.06)	0–14
Mental and behavioral disorders		F00-F99	12,468	1.9(1.45)	0–8	4,744	1.72(1.4)	0–8
	Organic, including symptomatic, mental disorders	F00-F09	8,015	1.22(1.17)	0–7	3,006	1.09(1.09)	0–7
	Mental and behavioral disorders due to psychoactive substance use	F10-F19	3,504	0.53(0.73)	0–5	1,408	0.51(0.73)	0–5
	Schizophrenia	F20	592	0.09(0.3)	0–2	203	0.07(0.27)	0–2
	Self-harm	X60-X84	25,476	3.87(2.69)	0–21	11,073	4.02(2.64)	0–18
Diseases of the digestive system		K00-K93	36,382	5.53(2.53)	0–17	14,688	5.33(2.47)	0–17
Diseases of the nervous system		G00-G99	10,295	1.57(1.37)	0–9	3,958	1.44(1.29)	0–9
Diseases of the genitourinary system		N00-N99	10,552	1.6(1.31)	0–8	4,257	1.55(1.28)	0–8
Diseases of the blood and blood-forming organs and certain disorders involving the immune mechanism		D50-D89	1,555	0.24(0.49)	0–3	673	0.24(0.5)	0–3

^a^ For warm season only (May to September)

^b^ Minimum-Maximum.

### Environmental Data

Measurements of relative humidity, air pressure, and ambient temperature from 1992 to 2009 were obtained from the Korea Meteorological Administration (KMA)[41]. Three-hour maximum temperature, mean relative humidity, and air pressure were measured from the representative synoptic surface observation station in Seoul (S1 Fig.). To control for potential confounding effects, 24-hour mean concentration of particulate matter (PM10) and 8-hour maximum concentration of ozone (O3) were obtained from the Korean National Institute of Environmental Research. Concentrations of air pollutants were measured every 15 min at 27 monitoring stations in Seoul.

### Statistical Analysis

Graphs that depict the relationship between temperature and mortality generally take the shape of a U, V, or J, and show an increase in mortality rate above a specific threshold temperature.[15, 23, 42, 43] The modeling approaches were adopted from well-established studies on air pollution [44, 45] and high ambient temperature.[15, 28, 43] An overdispersed Poisson generalized linear model (GLM) was used to analyze the relationship between mortality and daily maximum temperature from 1992 to 2009.[45, 46] PM10 (24-hour average), O3 level (daily maximum), relative humidity, day of the week, air pressure, and long-term time trends were selected as confounding variables. The threshold temperature was set at 90% of the maximum daily temperature during the study period, which was consistent with the findings from a previous study in Seoul.[11] S1 Table shows the number of days over the study period that had daily maximum temperatures higher than the threshold value. The analysis was based on the measurements taken from May to September of each year.

Daily attributable fractions and RRs were estimated using the threshold temperature (29.5°C) as the reference point, and then the total number of deaths attributable to high temperatures from 1992 to 2009 in Seoul were calculated.[19, 28] The attributable fraction of each disease on days higher than the threshold temperature was computed as follows:

Attributable fraction (AF) of deaths AF = (RR − 1)/RR, [19, 47]

The number of attributable deaths relative to the total number of deaths was also added to the result.

### Sensitivity Analyses

Sensitivity analyses were performed to explore the possible influences of PM10 and O3 on attributable deaths; however, this was only investigated for the 2001–2009 period (the only time period for which data were available; S1 Table). In addition, the RRs were compared using different lag days: single-day lag 1, lag 2, and moving average lag 0–2. The threshold temperature was also varied, being set at 93%, 95%, and 99% of the maximum daily temperature. All of these analyses were performed using SAS version 9.3 (SAS Institute Inc., USA) and R version 2.14 for Windows (http://cran.r-project.org/). All analyses were conducted in 2012–2013.

### Ethics Statement

This study qualified for exemption by the Institutional Review Board of the Graduate School of Public Health at Seoul National University. (IRB No. 65–2013–12–05)

## Results

During the study period, 271,633 (40%) of the 676,509 deaths occurred from May to September. Table 1 shows the descriptive statistics for cause-specific mortality in Seoul, based on ICD-10 codes. The main cause of death was cardiovascular disease (CVD). Table 2 summarizes the meteorological data. The average daily maximum temperature from May to September was 26.5°C, and 16.9°C for the full year. The pattern of daily all-cause mortality and temperature shows seasonal trends. All-cause mortality has a J-shaped relationship with temperature (S2 Fig.). The 1994 summer was the hottest, and there was some variation in each year. However, the average temperature remained stable (S3 Fig.). In 1994, temperature may have contributed to a higher number of deaths. Although there was temperature variation with regard to time and season, our statistical model controlled for the unmeasured time-varying potential confounder and showed the short-term temperature effect on mortality.

**Table 2:** Descriptive Statistics of meteorological variables (1992–2009) and air pollutants (2001–2009).

	All-season					Warm Season (May to September)				
	N(Days)	Mean	SD	Min.	Max.	N(Days)	Mean	SD	Min.	Max.
Daily Mean Temperature(°C)	6575	12.9	10.1	-15.7	33	2754	22.5	22.8	10.8	33
Daily Maximum Temperature(°C)	6575	16.9	10.3	-12.9	38.3	2754	26.5	26.8	12	38.3
Daily Minimum Temperature(°C)	6575	9.2	10.1	-18.4	28.9	2754	19	19.4	6.4	28.9
Daily Relative Humidity (%)	6575	62.9	14.5	18.8	97.4	2754	69.6	70.2	21.1	97.4
Daily Air pressure (hPA)	6575	1016.2	8.1	983.4	1040.6	2754	1009.3	1009.2	983.4	1025.7
Daily Maximum Ozone(ppm)	3867	33.9	19.9	2	143.2	1652	44.8	43.3	3.2	143.2
Daily Mean PM10(μg/m3)	3866	61.7	32.3	10	230.2	1652	52.7	47	10	206.7

**Fig 1:**
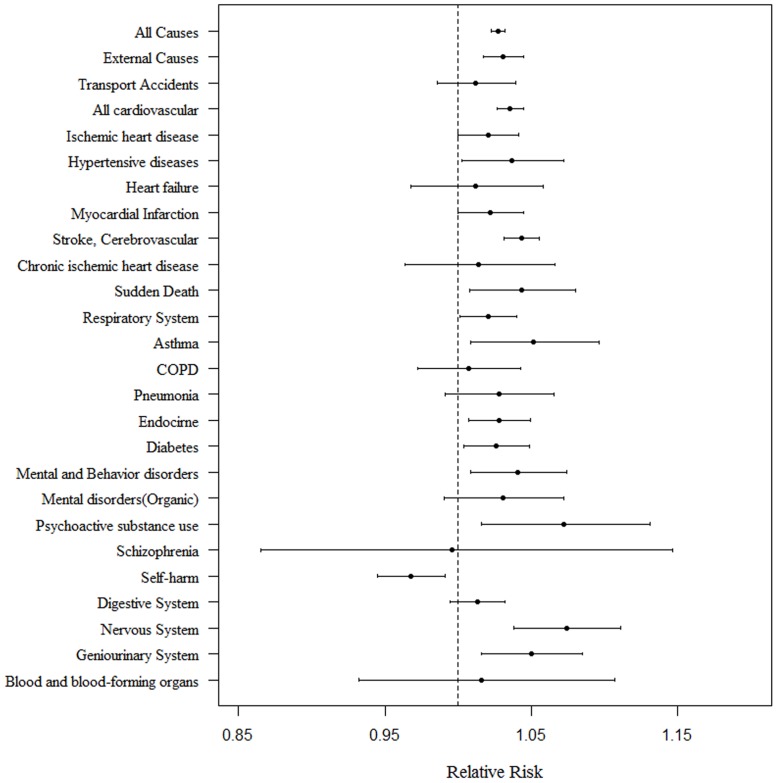
Estimated relative risk of mortality for every 1°C increase in temperature above the 29.5°C threshold from May through September, 1992–2009.

Fig. 1 displays the relations between daily maximum temperature and mortality. Above a threshold temperature of 29.5°C, a rise in temperature of 1°C resulted in an increase in death from all causes (RR 1.03; 95% confidence interval (CI) 1.02–1.03). This included cardiovascular conditions, (RR 1.04; 95% CI, 1.03–1.04); respiratory conditions (RR 1.02; 95% CI, 1–1.04); endocrine conditions (RR 1.03; 95% CI, 1.01–1.05); neurological conditions (RR 1.07; 95% CI, 1.04–1.11); genitourinary conditions (RR 1.05; 95% CI, 1.02–1.09); mental and behavioral disorders (RR 1.04; 95% CI, 1.01–1.07); and all accidental deaths (RR 1.03; 95% CI, 1.02–1.04). Analysis of the underlying causes of death showed a greater RR among mental and behavioral disorders due to psychoactive substance use (RR 1.07; 95% CI, 1.02–1.13). There was also an increased risk of death from asthma (RR 1.05, 95% CI, 1.01–1.11) and diabetes mellitus (1.03, 95% CI, 1–1.05).

Table 3 shows the estimated numbers and proportions of deaths attributable to high temperature from May to September. An estimated 3,177 (1.2%) of all deaths in the warm period of the year were due to high temperatures. This includes 975 of 65,675 (1.5%) deaths from cardiovascular conditions; 135 of 11,412 deaths (1.2%) from endocrine conditions; and 480 of 35,557 deaths (1.4%) from accidental causes. The greatest proportion of heat-attributable fatalities (3.3%) occurred with mental and behavioral disorders due to psychoactive substance use.

**Table 3:** Estimated number of deaths attributable to heat and estimated relative risk of mortality for a 1°C increase in temperature above the threshold temperature (29.5°C).

Main-category Diseases	Subcategory Diseases	Relative Risk	95% CI	Attributable Death(AD)	AD/ Death of specific cause(%)^a^	AD/Total Death(%)^b^
All cause		**1.03**	**(1.02–1.03)**	**3,177**	**1.17%**	**1.17%**
External causes of morbidity and mortality and injury(Accidental causes)		**1.03**	**(1.02–1.04)**	**480**	**1.35%**	**0.18%**
	Transport accidents	1.01	(0.99–1.04)	53	0.54%	0.02%
All cardiovascular		**1.04**	**(1.03–1.04)**	**975**	**1.48%**	**0.36%**
	Ischemic heart disease	1.02	(1–1.04)	105	0.83%	0.04%
	Hypertensive diseases	1.04	(1–1.07)	64	1.46%	0.02%
	Heart failure	1.01	(0.97–1.06)	13	0.46%	0.00%
	Myocardial Infarction	1.02	(1–1.04)	93	0.87%	0.03%
	Stroke, Cerebrovascular diseases	1.04	(1.03–1.06)	685	1.84%	0.25%
	Chronic ischemic heart disease	1.01	(0.96–1.07)	11	0.56%	0.00%
	Sudden Death	1.04	(1.01–1.08)	65	2.05%	0.02%
Respiratory System		**1.02**	**(1–1.04)**	**116**	**0.86%**	**0.04%**
	Asthma	1.05	(1.01–1.1)	54	2.10%	0.02%
	COPD	1.01	(0.97–1.04)	12	0.30%	0.00%
	Pneumonia	1.03	(0.99–1.07)	45	1.16%	0.02%
Endocrine, nutritional and metabolic diseases		**1.03**	**(1.01–1.05)**	**135**	**1.18%**	**0.05%**
	Diabetes mellitus	1.03	(1–1.05)	114	1.10%	0.04%
Mental and behavioral disorders		**1.04**	**(1.01–1.07)**	**87**	**1.83%**	**0.03%**
	Organic, including symptomatic, mental disorders	1.03	(0.99–1.07)	42	1.40%	0.02%
	Mental and behavioral disorders due to psychoactive substance use	1.07	(1.02–1.13)	46	3.27%	0.02%
	Schizophrenia	1	(0.86–1.15)	0	0.00%	0.00%
	Self-harm	0.97	(0.95–0.99)	-141	-1.27%	-0.05%
Diseases of the digestive system		**1.01**	**(0.99–1.03)**	**83**	**0.57%**	**0.03%**
Diseases of the nervous system		**1.07**	**(1.04–1.11)**	**121**	**3.06%**	**0.04%**
Diseases of the genitourinary system		**1.05**	**(1.02–1.09)**	**94**	**2.21%**	**0.03%**
Diseases of the blood and blood-forming organs and certain disorders involving the immune mechanism		**1.02**	**(0.93–1.11)**	**5**	**0.74%**	**0.00%**

^a^ Attributable death / Number of death from specific causes: Proportion of attributable death to each cause of death.

^b^ Attributable death / Total number of death: Proportion of attributable deaths to total number of death.

**Fig 2:**
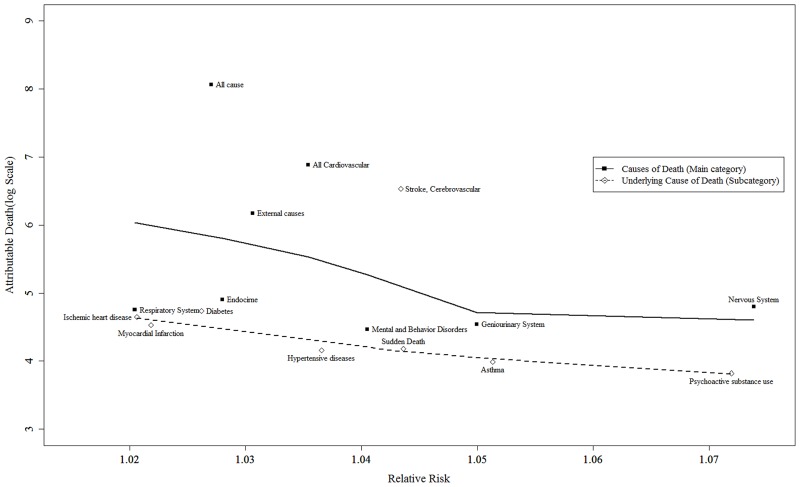
Relationships between relative risk and attributable deaths (log scale) for main-category and subcategory disease. Smoothed lines show the relationship between RRs and attributable deaths.

The RR and attributable deaths were compared, revealing that most heat-sensitive conditions (those with the highest RR) tended to be relatively uncommon causes of death, and the numbers of attributable deaths were less than for the major disease groups such as CVD (Fig. 2). The two LOWESS (locally weighted scatterplot smoothing) lines (span fraction = 1.0) of main-category and subcategory diseases were added. Both lines indicated a negative relationship between RRs and attributable deaths. Non-significant causes were excluded in this Fig.

### Sensitivity Analysis

Several additional analyses were performed to explore the sensitivity of the estimated RR to model specification. Specifically, sensitivity to 1) the inclusion of air pollutants, 2) exposure lag, and 3) threshold temperature were examined.

In addition to the adverse health effects of high temperatures, measured air pollution variables should be considered. In this study, adjusting air pollution in the model yielded nearly the same estimates as did the non-adjusted model (S2 Table). Consequently, the models used in this study are believed to have adequately captured the main effects of temperature on mortality.

The lag in the time series models was also changed to single lag 1, 2, and moving average lag 0–2 days, which yielded similar results (S3 Table). For all-cause deaths, the estimated attributable deaths of lag 1 was the largest. The lag 1 estimates for each disease showed a pattern similar to that of lag 0, but were slightly larger. Estimates for endocrine, nervous, and genitourinary system conditions were slightly larger for lag 0.

In addition, four different temperatures, which corresponded to the 90th, 93th, 95th, and 99th percentiles for daily maximum temperature during the 19 years, were compared. The number of days over the study period where the maximum temperature was higher than the threshold value is shown in S1 Table. When the threshold temperature increases, the RR estimates increase (S4 Table). Although the RR increases, the corresponding days in which the temperature is higher than the threshold decreases, so the number of attributable days appears to decrease when the threshold increases.

## Discussion

Several previous epidemiological studies have reported a correlation between temperature changes and disease-related mortality. In this study, the RRs above the threshold temperature and the number of heat-attributable deaths for each type of disease from 1992 to 2009 were calculated, particularly including sub-categories of the major disease groups. Results showed that almost all diseases were associated with a greater mortality risk during periods of unusual heat. The proportion of deaths attributable to days with a maximum temperature greater than 29.5 degrees ranged from 0.83% to 3.27%.

The present investigation builds on previous work on mortality in Seoul, and may be the first study conducted in an East Asian metropolitan city that examined the effects of heat on disease-specific mortality. Several studies that were conducted in cities in Europe and the US have identified specific populations vulnerable to mortality during high ambient temperatures.[18, 19, 24, 48] The present study found that high temperature was linked with increases in mortality for most primary causes of death, including cardiovascular, respiratory, endocrine, nutritional, metabolic, neurological, genitourinary, digestive, mental, and behavioral disorders.

The observations herein are similar to those reported previously on the relationship between total mortality and ambient temperature. In this study, the results indicated that the overall mortality risk increased by 2.7% for every 1°C increase above threshold temperature. In a study conducted in Wales and 10 regions of London, total mortality risk increased by 2.1% (95% CI 1.6–2.6) for every 1°C increase in threshold temperature.[19] A study conducted by Chung et al. in Seoul reported a 2.7% increase (95% CI, 2.2–3.1) in daily non-accidental mortality, using a threshold temperature of 30.1–33.5°.[11] Note that this earlier study in Seoul used a different temperature metric (apparent temperature), included all seasons of the year, and was based on a shorter period of observation (1991–2006); however, the present results are still similar.

Also similar to previous reports, the present results show that the mortality rate from cardiovascular conditions increases with high ambient temperatures (3.5%; 95% CI, 2.6–4.5). Specific cardiovascular causes of death that are significantly affected by high temperatures include sudden death (4.4%), cerebrovascular diseases (4.3%), hypertensive diseases (3.7%), and ischemic heart disease (2.1%).

Data on the relationship between the mortality rate from respiratory conditions and high ambient temperatures have been inconsistent. Several studies reported no significant change in the number of respiratory deaths as a function of high temperature during the summer or in hot cities.[6, 11, 48, 49] The present results do show that total respiratory mortality increases when the temperature rises, but an effect of high temperature on mortality due to pneumonia and chronic obstructive pulmonary disease (COPD) was not found in the present study. However, it is clear that deaths from asthma were sensitive to high temperatures.

The results of this study reveal a relatively high increase in the RR of mortality from neurological (7.4%) and mental and behavioral disorders (4.1%). This increase in mortality may occur when extremely high temperatures induce neurological dysfunction and multi-organ failure.[8, 9, 50–53] However, other risk factors cannot be overlooked. It is well-known that patients taking antipsychotic or psychotropic medications may be vulnerable to high temperatures, and the health effects observed in this study might be partially drug-related.[3, 9, 53–55] Additionally, a significant heat-associated increase in the RR of mortality from genitourinary conditions was observed (5%). Thus, the present results show that patients with a wide range of pre-existing chronic conditions may be more susceptible to high ambient temperatures.

Unlike previous studies that focused solely on identifying the RRs of highly prevalent diseases, the present study determined the RRs and attributable deaths for ICD-10 subcategory diseases. Some of these conditions, especially asthma and psychoactive-substance-use-related mental disorders, were found to be particularly heat sensitive. Furthermore, there was a negative relationship between RR and attributable death (log scale) in this study. The most heat-sensitive diseases (those with the highest RRs) were not the diseases that caused the largest numbers of deaths attributable to high temperatures.

There were several limitations of the present study. First, the maximum daily temperature (lag 0) was used in the final model. Although sensitivity analyses using different lag days (single lag 1, single lag 2, and moving average lag 0–2) were performed, the results did not substantially change (S3 Table). High ambient temperatures generally had an immediate effect, but it is possible that lag patterns differ between diseases.[26, 56]

Second, threshold temperature used herein corresponded to the 90th percentile of the maximum daily temperature, which has been used previously.[11, 19, 57] In Japan, researchers examined the optimum temperature at which mortality is the lowest, observing that this point was 80–85% of the daily maximum temperature.[57] However, there are alternate methods of estimating the threshold temperature. [27, 58, 59] One of the most commonly used methods is grid search. It searches for the point that minimizes deviation among figures of the simulated model. Some studies compare complex models that assume a non-linear relationship between mortality and temperature.[23, 28] Depending on the method used, the observed heat effect can vary. Moreover, the threshold temperature may differ for each disease because the underlying mechanisms by which temperature influences mortality is likely vary by disease. Despite this, using the percentage distribution method has been reported as effective and having advantages compared with alternate methods and their outcomes.[28, 56, 57, 60] The sensitivity analysis results on different percentiles of the maximum daily temperature were included (S4 Table). There were no substantial change in the results between the 93^rd^ and 95^th^ percentiles.

Third, the effect of air pollutants for the entire study period could not be analyzed because air pollution data were not available until 2001. To estimate how the missing information on air pollutants may have affected the results another sensitivity analysis was conducted, after adjusting for this information—the results of the adjusted model and unadjusted model were robust (S2 Table). Thus, air pollution did not appear to be a concern.

Finally, despite the consistency in the results, there may be some contextual variability in the effects of temperature on mortality across locations within Seoul and among different populations, levels of social development, and individual sensitivities and adaptive capacities. Due to the urban heat island effect, there is also temperature variability within a metropolitan region that is not measured in this study because temperature was recorded at only one weather station.

## Conclusion

This study estimated the RRs and attributable deaths related to high ambient temperature and included a wide variety of diseases. The present results indicate that high ambient temperature increases mortality for many conditions and that the effect varies with each disease. Some relatively uncommon conditions—that is, those with a low number of attributable deaths—appear to be highly sensitive to heat, warranting close attention by interventions that are designed to manage the risk of high temperature. In this, as in other areas of public health, policymakers must consider both vulnerable sub-groups and the predominant causes of disease burden.

## Supporting Information

S1 Fig Map of study area. Seoul divided into 25 gu (districts) that vary greatly in area (from 10 to 27 km^2^) and population. Map color indicates population density. Smallest: 726 person/km^2^; largest: 29,104 person/km^2^.
(TIF)

S2 Fig The association between daily maximum temperature and mortality during the 18 year study period, 1992–2009 (full calendar year). (a) Time-series plot of mortality from 1992 to2009. (b) Time-series plot of temperature from 1992 to 2009. (c) Daily all causes mortality in Seoul, and relationships between observed maximum temperature and daily mortality for full calendar year. The figures on the right show a “J” shape with increases in mortality at certain temperatures.
(TIF)

S3 Fig Box plot for annual average maximum temperature from 1992 to 2009. (a) Full calendar year average. (b) May to September average. The 1994 and 2004 summers were the hottest, and there was some variation within each year.
(TIF)

S1 Table Maximum Temperatures at 75%, 80%, 85%, 90%, 95%, 99% and overall day counts above the temperature (1992~2009). (DOCX)

S2 Table Sensitivity Analysis: Model comparison using PM10 and Ozone (Study Period: 2001–2009). (DOCX)

S3 Table Sensitivity Analysis: Model comparison using different lag days (Single Day lag0, Single day lag 2, and Moving average lag 0–2). (DOCX)

S4 Table Sensitivity analysis: Model comparison using different threshold temperature point. (DOCX)

## References

[1] Kovats RS, Kristie LE. Heatwaves and public health in Europe. Eur J Public Health. 2006;16(6):592–9. Epub 2006/04/29. 16644927 10.1093/eurpub/ckl049

[2] Semenza JC, Rubin CH, Falter KH, Selanikio JD, Flanders WD, Howe HL, et al Heat-related deaths during the July 1995 heat wave in Chicago. N Engl J Med. 1996;335(2):84–90. Epub 1996/07/11. 8649494 10.1056/NEJM199607113350203

[3] Koppe C, Kovats S, Jendritzky G, Menne B, Breuer DJ, Wetterdienst D. Heat waves: risks and responses: Regional Office for Europe, World Health Organization; 2004.

[4] Luber G, McGeehin M. Climate change and extreme heat events. American journal of preventive medicine. 2008;35(5):429–35. Epub 2008/10/22. 10.1016/j.amepre.2008.08.021 18929969

[5] Abrignani MG, Corrao S, Biondo GB, Renda N, Braschi A, Novo G, et al Influence of climatic variables on acute myocardial infarction hospital admissions. International journal of cardiology. 2009;137(2):123–9. 10.1016/j.ijcard.2008.06.036 18694607

[6] Braga AL, Zanobetti A, Schwartz J. The effect of weather on respiratory and cardiovascular deaths in 12 U.S. cities. Environ Health Perspect. 2002;110(9):859–63. 12204818 10.1289/ehp.02110859 PMC1240983

[7] Turner LR, Barnett AG, Connell D, Tong S. Ambient temperature and cardiorespiratory morbidity: a systematic review and meta-analysis. Epidemiology. 2012;23(4):594–606. Epub 2012/04/26. 10.1097/EDE.0b013e3182572795 22531668

[8] Bouchama A, Knochel JP. Heat stroke. N Engl J Med. 2002;346(25):1978–88. 12075060 10.1056/NEJMra011089

[9] Bouchama A, Dehbi M, Mohamed G, Matthies F, Shoukri M, Menne B. Prognostic factors in heat wave related deaths: a meta-analysis. Archives of internal medicine. 2007;167(20):2170–6. 17698676 10.1001/archinte.167.20.ira70009

[10] Hajat S, O'Connor M, Kosatsky T. Health effects of hot weather: from awareness of risk factors to effective health protection. Lancet. 2010;375(9717):856–63. Epub 2010/02/16. 10.1016/S0140-6736(09)61711-6 20153519

[11] Chung JY, Honda Y, Hong YC, Pan XC, Guo YL, Kim H. Ambient temperature and mortality: an international study in four capital cities of East Asia. The Science of the total environment. 2009;408(2):390–6. 10.1016/j.scitotenv.2009.09.009 19853280

[12] Hajat S, Armstrong BG, Gouveia N, Wilkinson P. Mortality Displacement of Heat-Related Deaths: A Comparison of Delhi, Sao Paulo, and London. Epidemiology. 2005;16(5):613–20. 16135936 10.1097/01.ede.0000164559.41092.2a

[13] Basu R. High ambient temperature and mortality: a review of epidemiologic studies from 2001 to 2008. Environ Health. 2009;8:40 Epub 2009/09/18. 10.1186/1476-069X-8-40 19758453 PMC2759912

[14] Gosling S, Lowe J, McGregor G, Pelling M, Malamud B. Associations between elevated atmospheric temperature and human mortality: a critical review of the literature. Climatic Change. 2009;92(3–4):299–341. 10.1007/s10584-008-9441-x

[15] Armstrong B. Models for the relationship between ambient temperature and daily mortality. Epidemiology. 2006;17(6):624–31. Epub 2006/10/10. 17028505 10.1097/01.ede.0000239732.50999.8f

[16] Basu R, Samet JM. Relation between Elevated Ambient Temperature and Mortality: A Review of the Epidemiologic Evidence. Epidemiologic Reviews. 2002;24(2):190–202. 12762092 10.1093/epirev/mxf007

[17] Rey G, Jougla E, Fouillet A, Pavillon G, Bessemoulin P, Frayssinet P, et al The impact of major heat waves on all-cause and cause-specific mortality in France from 1971 to 2003. International archives of occupational and environmental health. 2007;80(7):615–26. 17468879 10.1007/s00420-007-0173-4 PMC2291483

[18] Basagana X, Sartini C, Barrera-Gomez J, Dadvand P, Cunillera J, Ostro B, et al Heat waves and cause-specific mortality at all ages. Epidemiology. 2011;22(6):765–72. 10.1097/EDE.0b013e31823031c5 21968768

[19] Gasparrini A, Armstrong B, Kovats S, Wilkinson P. The effect of high temperatures on cause-specific mortality in England and Wales. Occup Environ Med. 2012;69(1):56–61. Epub 2011/03/11. 10.1136/oem.2010.059782 21389012

[20] O'Neill MS, Zanobetti A, Schwartz J. Modifiers of the temperature and mortality association in seven US cities. American journal of epidemiology. 2003;157(12):1074–82. 12796043 10.1093/aje/kwg096

[21] Zanobetti A, Schwartz J. Temperature and Mortality in Nine US Cities. Epidemiology. 2008;19(4):563–70. 10.1097/EDE.0b013e31816d652d 18467963 PMC3722554

[22] Basu R, Dominici F, Samet JM. Temperature and Mortality Among the Elderly in the United States: A Comparison of Epidemiologic Methods. Epidemiology. 2005;16(1):58–66. 15613946 10.1097/01.ede.0000147117.88386.fe

[23] Hajat S, Armstrong B, Baccini M, Biggeri A, Bisanti L, Russo A, et al Impact of high temperatures on mortality: is there an added heat wave effect? Epidemiology. 2006;17(6):632–8. Epub 2006/09/28. 17003686 10.1097/01.ede.0000239688.70829.63

[24] Stafoggia M, Forastiere F, Agostini D, Biggeri A, Bisanti L, Cadum E, et al Vulnerability to Heat-Related Mortality: A Multicity, Population-Based, Case-Crossover Analysis. Epidemiology. 2006;17(3):315–23. 16570026 10.1097/01.ede.0000208477.36665.34

[25] Baccini M, Biggeri A, Accetta G, Kosatsky T, Katsouyanni K, Analitis A, et al Heat effects on mortality in 15 European cities. Epidemiology. 2008;19(5):711–9. Epub 2008/06/04. 10.1097/EDE.0b013e318176bfcd 18520615

[26] Curriero FC, Heiner KS, Samet JM, Zeger SL, Strug L, Patz JA. Temperature and Mortality in 11 Cities of the Eastern United States. American journal of epidemiology. 2002;155(1):80–7. 11772788 10.1093/aje/155.1.80

[27] Ulm K. A statistical method for assessing a threshold in epidemiological studies. Statistics in medicine. 1991;10(3):341–9. 2028118 10.1002/sim.4780100306

[28] Armstrong BG, Chalabi Z, Fenn B, Hajat S, Kovats S, Milojevic A, et al Association of mortality with high temperatures in a temperate climate: England and Wales. J Epidemiol Community Health. 2011;65(4):340–5. Epub 2010/05/05. 10.1136/jech.2009.093161 20439353

[29] Campbell-Lendrum D, Woodruff R. Comparative Risk Assessment of the Burden of Disease from Climate Change. Environmental Health Perspectives. 2006;114(12):1935–41. 17185288 10.1289/ehp.8432 PMC1764135

[30] Kovats RS, Campbell-Lendrum D, Matthies F. Climate Change and Human Health: Estimating Avoidable Deaths and Disease. Risk Analysis. 2005;25(6):1409–18. 16506971 10.1111/j.1539-6924.2005.00688.x

[31] Geneva: World Health Organization; 2004 Global climate change. In Comparative Quantification of Health Risks; p. 1543–649.

[32] Levin ML. The occurrence of lung cancer in man. Acta—Unio Internationalis Contra Cancrum. 1953;9(3):531–41. PubMed PMID: 13124110. 13124110

[33] Mokdad AH, Marks JS, Stroup DF, Gerberding JL. Actual causes of death in the United States, 2000. JAMA: the journal of the American Medical Association. 2004;291(10):1238–45. 15010446 10.1001/jama.291.10.1238

[34] Mathers C, Stevens G, Mascarenhas M. Global health risks: mortality and burden of disease attributable to selected major risks: World Health Organization; 2009.

[35] Statistics Korea 2013 [updated 2013–09–17]. Available from: www.kostat.go.kr.

[36] Lau K-M, Li M-T. The Monsoon of East Asia and its Global Associations—A Survey. Bulletin of the American Meteorological Society. 1984;65(2):114–25. 10.1175/1520-0477(1984)065<0114:TMOEAA>2.0.CO;2

[37] Yihui D, Chan JCL. The East Asian summer monsoon: an overview. Meteorol Atmos Phys. 2005;89(1–4):117–42. 10.1007/s00703-005-0125-z

[38] Tran H, Uchihama D, Ochi S, Yasuoka Y. Assessment with satellite data of the urban heat island effects in Asian mega cities. International Journal of Applied Earth Observation and Geoinformation. 2006;8(1):34–48. 10.1016/j.jag.2005.05.003

[39] Yoon SJ, Lee SJ, Hong YD, Song CK, Yu JA, Kim SY. Korean Climate Change Assessment Report 2010. National Institute of Environmental Research,Ministry of Environment, Korea; 2010.

[40] Parry ML. Climate Change 2007: Impacts, Adaptation and Vulnerability: Working Group I Contribution to the Fourth Assessment Report of the IPCC: Cambridge University Press; 2007.

[41] Korea Meteorological Administration 2013 [cited 2013]. Available from: http://www.kma.go.kr/.

[42] Curriero FC, Heiner KS, Samet JM, Zeger SL, Strug L, Patz JA. Temperature and mortality in 11 cities of the eastern United States. American journal of epidemiology. 2002;155(1):80–7. 11772788 10.1093/aje/155.1.80

[43] Pattenden S, Nikiforov B, Armstrong BG. Mortality and temperature in Sofia and London. J Epidemiol Community Health. 2003;57(8):628–33. 12883072 10.1136/jech.57.8.628 PMC1732532

[44] Dominici F, Sheppard L, Clyde M. Health Effects of Air Pollution: A Statistical Review. International Statistical Review. 2003;71(2):243–76. 10.1111/j.1751-5823.2003.tb00195.x

[45] Peng RD, Dominici F, Louis TA. Model choice in time series studies of air pollution and mortality. J Roy Stat Soc a Sta. 2006;169:179–98. 10.1111/j.1467-985X.2006.00410.x. PubMed PMID: WOS:000235333700001

[46] MacCullagh P, Nelder JA. Generalized linear models: CRC press; 1989.

[47] Walter SD. The Estimation and Interpretation of Attributable Risk in Health Research. Biometrics. 1976;32(4):829–49. 1009228

[48] Basu R, Ostro BD. A multicounty analysis identifying the populations vulnerable to mortality associated with high ambient temperature in California. American journal of epidemiology. 2008;168(6):632–7. 10.1093/aje/kwn170 18663214

[49] Kilbourne EM. The spectrum of illness during heat waves. American journal of preventive medicine. 1999;16(4):359–60. Epub 1999/09/24. 10493296 10.1016/s0749-3797(99)00016-1

[50] Leon LR, Helwig BG. Heat stroke: Role of the systemic inflammatory response. Journal of Applied Physiology. 2010;109(6):1980–8. 10.1152/japplphysiol.00301.2010 20522730

[51] Yaqub B, Al Deeb S. Heat strokes: aetiopathogenesis, neurological characteristics, treatment and outcome. Journal of the Neurological Sciences. 1998;156(2):144–51. 10.1016/S0022-510X(98)00037-9 9588849

[52] Hansen AL, Bi P, Ryan P, Nitschke M, Pisaniello D, Tucker G. The effect of heat waves on hospital admissions for renal disease in a temperate city of Australia. International journal of epidemiology. 2008;37(6):1359–65. 10.1093/ije/dyn165 18710886

[53] Semenza JC. Acute renal failure during heat waves. American journal of preventive medicine. 1999;17(1):97 10429760 10.1016/s0749-3797(99)00066-5

[54] Hansen A, Bi P, Nitschke M, Ryan P, Pisaniello D, Tucker G. The effect of heat waves on mental health in a temperate Australian city. Environ Health Perspect. 2008;116(10):1369–75. 10.1289/ehp.11339 18941580 PMC2569097

[55] Stöllberger C, Lutz W, Finsterer J. Heat-related side-effects of neurological and non-neurological medication may increase heatwave fatalities. European Journal of Neurology. 2009;16(7):879–82. 10.1111/j.1468-1331.2009.02581.x 19453697

[56] Anderson BG, Bell ML. Weather-related mortality: how heat, cold, and heat waves affect mortality in the United States. Epidemiology. 2009;20(2):205–13. 10.1097/EDE.0b013e318190ee08 19194300 PMC3366558

[57] Honda Y, Kabuto M, Ono M, Uchiyama I. Determination of optimum daily maximum temperature using climate data. Environmental health and preventive medicine. 2007;12(5):209–16. 10.1265/ehpm.12.209 21432083 PMC2723377

[58] Muggeo VMR. Estimating regression models with unknown break-points. Statistics in medicine. 2003;22(19):3055–71. 12973787 10.1002/sim.1545

[59] Lerman PM. Fitting Segmented Regression Models by Grid Search. Journal of the Royal Statistical Society Series C (Applied Statistics). 1980;29(1):77–84. 10.2307/2346413

[60] Pascal M, Wagner V, Le Tertre A, Laaidi K, Honoré C, Bénichou F, et al Definition of temperature thresholds: the example of the French heat wave warning system. International journal of biometeorology. 2013;57(1):21–9. 10.1007/s00484-012-0530-1 22361805

