# Supplementary figures and images for: Heat-Attributable Deaths between 1992 and 2009 in Seoul, South Korea
Source: PLoS One. 2015 Feb 18;10(2):e0118577. doi: 10.1371/journal.pone.0118577 (PMC4334895; doi:10.1371/journal.pone.0118577)

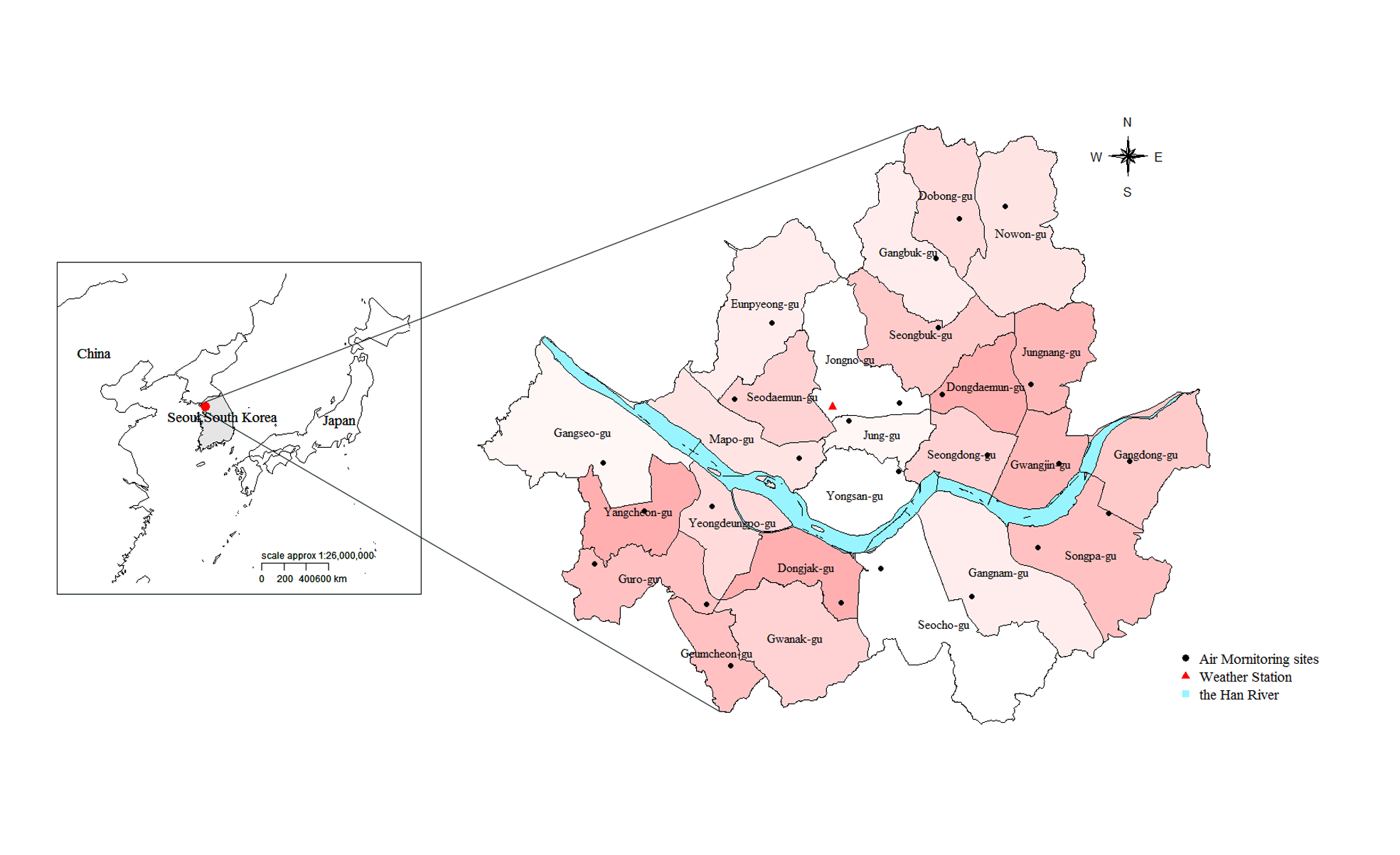

Supplement: S1 Fig — Seoul divided into 25 gu (districts) that vary greatly in area (from 10 to 27 km2) and population. Map color indicates population density. Smallest: 726 person/km2; largest: 29,104 person/km2. (TIF) [file pone.0118577.s001.tif]

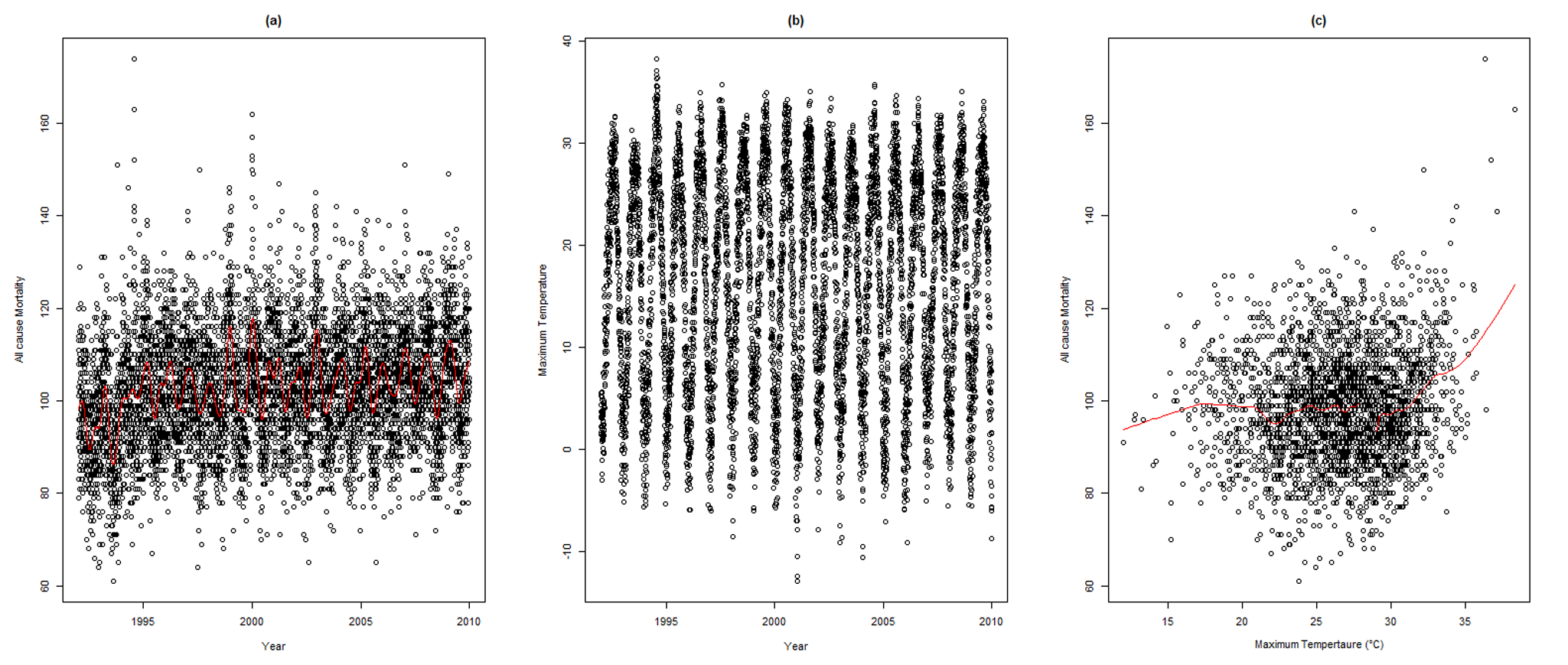

Supplement: S2 Fig — (a) Time-series plot of mortality from 1992 to2009. (b) Time-series plot of temperature from 1992 to 2009. (c) Daily all causes mortality in Seoul, and relationships between observed maximum temperature and daily mortality for full calendar year. The figures on the right show a “J” shape with increases in mortality at certain temperatures. (TIF) [file pone.0118577.s002.tif]

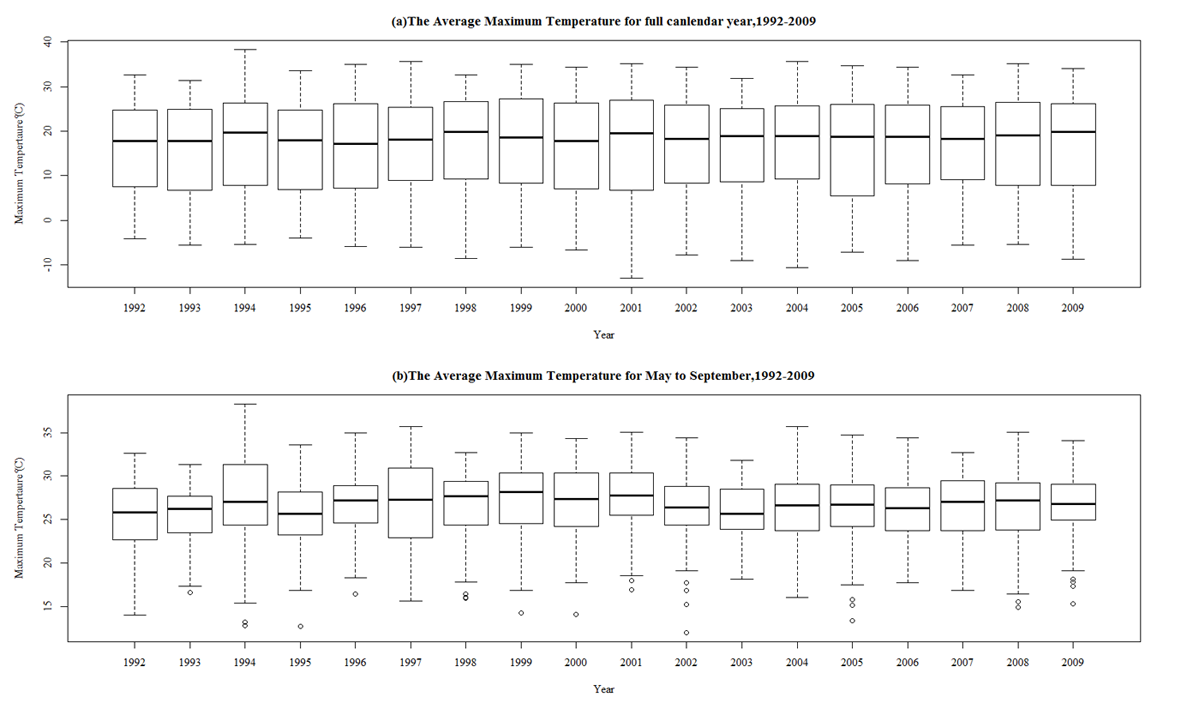

Supplement: S3 Fig — (a) Full calendar year average. (b) May to September average. The 1994 and 2004 summers were the hottest, and there was some variation within each year. (TIF) [file pone.0118577.s003.tif]
