# Supplementary material for: Heat-Attributable Deaths between 1992 and 2009 in Seoul, South Korea
Source: PLoS One. 2015 Feb 18;10(2):e0118577. doi: 10.1371/journal.pone.0118577 (PMC4334895; doi:10.1371/journal.pone.0118577)
Supplement: S1 Table — (DOCX) [file pone.0118577.s004.docx]

S1 Table. Maximum Temperatures at 75%, 80%, 85%, 90%, 95%, 99% and overall day counts above the temperature (1992~2009)

| Study Duration | Total days | 75% | 80% | 85% | 90% | 93% | 95% | 99% |
| --- | --- | --- | --- | --- | --- | --- | --- | --- |
| 1992-2009 | 6575 days | 25.9°C | 27.1°C | 28.20°C | 29.5°C | 30.3°C | 31°C | 33.4°C |
|  |  | 1639days | 1284days | 968days | 624days | 448days | 309days | 64days |
| 2001-2009 | 3287days | 25.95°C | 27.0°C | 28.1°C | 29.2°C | 30.0°C | 30.67°C | 33.0°C |
|  |  | 822days | 649days | 492days | 326days | 220days | 165days | 26days |
