# Supplementary material for: Heat-Attributable Deaths between 1992 and 2009 in Seoul, South Korea
Source: PLoS One. 2015 Feb 18;10(2):e0118577. doi: 10.1371/journal.pone.0118577 (PMC4334895; doi:10.1371/journal.pone.0118577)
Supplement: S2 Table — (DOCX) [file pone.0118577.s005.docx]

S2 Table. Sensitivity Analysis: Model comparison using PM10 and Ozone (Study Period: 2001-2009)

|  | **Adjusted Model** | | |  | **Non-adjusted Model** | | |
| --- | --- | --- | --- | --- | --- | --- | --- |
|  | **RR** | **95% CI** | **AD** |  | **RR** | **95% CI** | **AD** |
| **All cause** | **1.02** | **(1.01-1.03)** | **1104** |  | **1.02** | **(1.01-1.02)** | **1010** |
| **External causes of morbidity and mortality and injury** | **1.03** | **(1.01-1.05)** | **200** |  | **1.02** | **(1.01-1.04)** | **172** |
| Transport accidents | 1.03 | (0.99-1.07) | 38 |  | 1.03 | (0.99-1.07) | 43 |
| **All cardiovascular** | **1.02** | **(1-1.03)** | **207** |  | **1.01** | **(1-1.03)** | **170** |
| Ischemic heart disease | 1.02 | (0.99-1.04) | 53 |  | 1.01 | (0.98-1.04) | 29 |
| Hypertensive diseases | 1.02 | (0.98-1.07) | 25 |  | 1.02 | (0.97-1.07) | 19 |
| Heart failure | 0.98 | (0.91-1.06) | -8 |  | 0.99 | (0.92-1.06) | -6 |
| Myocardial Infarction | 1.01 | (0.98-1.04) | 33 |  | 1.01 | (0.98-1.04) | 19 |
| Stroke, Cerebrovascular diseases | 1.02 | (1-1.04) | 151 |  | 1.02 | (1-1.03) | 122 |
| Chronic ischemic heart disease | 1.04 | (0.97-1.11) | 18 |  | 1.02 | (0.96-1.09) | 10 |
| Sudden Death | 1.00 | (0.93-1.08) | 0 |  | 1.01 | (0.94-1.08) | 4 |
| **Respiratory System** | **1.01** | **(0.98-1.03)** | **14** |  | **1.00** | **(0.97-1.03)** | **-8** |
| Asthma | 1.08 | (1.01-1.16) | 36 |  | 1.07 | (1-1.14) | 30 |
| COPD | 0.95 | (0.9-1.01) | -41 |  | 0.95 | (0.9-1) | -46 |
| Pneumonia | 1.01 | (0.96-1.07) | 12 |  | 1.00 | (0.95-1.06) | 2 |
| **Endocrine, nutritional and metabolic diseases** | **1.02** | **(0.99-1.05)** | **46** |  | **1.01** | **(0.99-1.04)** | **36** |
| Diabetes mellitus | 1.02 | (0.99-1.05) | 49 |  | 1.02 | (0.99-1.05) | 46 |
| **Mental and behavioral disorders** | **1.05** | **(1-1.1)** | **54** |  | **1.04** | **(1-1.09)** | **45** |
| Organic, including symptomatic, mental disorders | 1.03 | (0.97-1.09) | 21 |  | 1.02 | (0.97-1.08) | 17 |
| PSU | 1.1 | (1.01-1.2) | 28 |  | 1.09 | (1-1.18) | 25 |
| Schizophrenia | 1.03 | (0.8-1.33) | 1 |  | 1.00 | (0.81-1.24) | 0 |
| Self-harm | 0.98 | (0.95-1.01) | -48 |  | 0.98 | (0.95-1.01) | -63 |
| **Diseases of the digestive system** | **1.03** | **(1-1.06)** | **91** |  | **1.04** | **(1.01-1.07)** | **98** |
| **Diseases of the nervous system** | **1.05** | **(1-1.1)** | **50** |  | **1.04** | **(1-1.09)** | **44** |
| **Diseases of the genitourinary system** | **1.02** | **(0.97-1.07)** | **17** |  | **1.03** | **(0.99-1.08)** | **35** |
| **Diseases of the blood and blood-forming organs and certain disorders involving the immune mechanism** | **1.02** | **(0.9-1.16)** | **3** |  | **1.04** | **(0.92-1.17)** | **5** |
