# Supplementary material for: Heat-Attributable Deaths between 1992 and 2009 in Seoul, South Korea
Source: PLoS One. 2015 Feb 18;10(2):e0118577. doi: 10.1371/journal.pone.0118577 (PMC4334895; doi:10.1371/journal.pone.0118577)
Supplement: S3 Table — (DOCX) [file pone.0118577.s006.docx]

S3 Table. Sensitivity Analysis: Model comparison using different lag days (Single Day lag0, Single day lag 2, and Moving average lag 0-2)

|  | **Single Day Lag 1** | | |  | **Single Day Lag 2** | | |  | **Moving Average Lag 0-2** | | |
| --- | --- | --- | --- | --- | --- | --- | --- | --- | --- | --- | --- |
|  | **RR** | **95% CI** | **AD** |  | **RR** | **95% CI** | **AD** |  | **RR** | **95% CI** | **AD** |
| **All cause** | **1.03** | **(1.02-1.03)** | **3248** |  | **1.02** | **(1.02-1.02)** | **2347** |  | **1.03** | **(1.03-1.04)** | **3068** |
| **External causes of morbidity and mortality and injury** | **1.03** | **(1.02-1.04)** | **485** |  | **1.02** | **(1-1.03)** | **257** |  | **1.03** | **(1.01-1.05)** | **370** |
| Transport accidents | 1.01 | (0.98-1.03) | 26 |  | 0.99 | (0.96-1.01) | -56 |  | 0.99 | (0.96-1.02) | -51 |
| **All cardiovascular** | **1.04** | **(1.03-1.05)** | **1070** |  | **1.03** | **(1.02-1.04)** | **928** |  | **1.05** | **(1.04-1.06)** | **1068** |
| Ischemic heart disease | 1.02 | (1-1.04) | 110 |  | 1.01 | (0.99-1.03) | 71 |  | 1.03 | (1-1.05) | 97 |
| Hypertensive diseases | 1.04 | (1.01-1.08) | 79 |  | 1.06 | (1.02-1.09) | 100 |  | 1.07 | (1.03-1.11) | 89 |
| Heart failure | 1.04 | (0.99-1.08) | 42 |  | 1.01 | (0.97-1.06) | 11 |  | 1.04 | (0.98-1.09) | 30 |
| Myocardial Infarction | 1.02 | (0.99-1.04) | 70 |  | 1.01 | (0.99-1.03) | 44 |  | 1.02 | (1-1.05) | 76 |
| Stroke, Cerebrovascular diseases | 1.05 | (1.03-1.06) | 730 |  | 1.04 | (1.03-1.05) | 655 |  | 1.06 | (1.05-1.07) | 736 |
| Chronic ischemic heart disease | 1.05 | (1-1.1) | 38 |  | 1.03 | (0.98-1.08) | 26 |  | 1.03 | (0.98-1.1) | 21 |
| Sudden Death | 1.06 | (1.03-1.1) | 93 |  | 1.05 | (1.02-1.09) | 80 |  | 1.07 | (1.03-1.11) | 81 |
| **Respiratory System** | **1.04** | **(1.02-1.05)** | **199** |  | **1.02** | **(1-1.04)** | **123** |  | **1.04** | **(1.02-1.06)** | **164** |
| Asthma | 1.08 | (1.04-1.13) | 87 |  | 1.03 | (0.99-1.07) | 32 |  | 1.08 | (1.03-1.13) | 61 |
| COPD | 1.04 | (1-1.07) | 65 |  | 1.02 | (0.99-1.06) | 41 |  | 1.03 | (0.99-1.07) | 36 |
| Pneumonia | 1.03 | (0.99-1.07) | 47 |  | 1.03 | (0.99-1.06) | 44 |  | 1.04 | (1-1.09) | 52 |
| **Endocrine, nutritional and metabolic diseases** | **1.03** | **(1-1.05)** | **121** |  | **1.01** | **(0.99-1.03)** | **40** |  | **1.03** | **(1.01-1.06)** | **111** |
| Diabetes mellitus | 1.03 | (1-1.05) | 114 |  | 1.01 | (0.99-1.03) | 39 |  | 1.03 | (1.01-1.06) | 104 |
| **Mental and behavioral disorders** | **1.05** | **(1.02-1.08)** | **109** |  | **1.03** | **(1-1.06)** | **59** |  | **1.05** | **(1.01-1.09)** | **85** |
| Organic, including symptomatic, mental disorders | 1.05 | (1.01-1.1) | 72 |  | 1.03 | (0.99-1.07) | 37 |  | 1.05 | (1-1.1) | 51 |
| PSU | 1.03 | (0.98-1.09) | 20 |  | 1.01 | (0.95-1.07) | 5 |  | 1.05 | (0.98-1.11) | 23 |
| Schizophrenia | 1.18 | (1.04-1.33) | 15 |  | 1.17 | (1.03-1.33) | 14 |  | 1.15 | (1-1.33) | 11 |
| Self-harm | 0.98 | (0.96-1) | -92 |  | 0.98 | (0.96-1.01) | -72 |  | 0.97 | (0.95-1) | -91 |
| **Diseases of the digestive system** | **1.02** | **(1-1.04)** | **103** |  | **1.01** | **(0.99-1.03)** | **79** |  | **1.02** | **(1-1.04)** | **89** |
| **Diseases of the nervous system** | **1.04** | **(1-1.08)** | **66** |  | **1.03** | **(1-1.07)** | **56** |  | **1.07** | **(1.03-1.12)** | **91** |
| **Diseases of the genitourinary system** | **1.02** | **(0.98-1.05)** | **35** |  | **1.00** | **(0.97-1.04)** | **5** |  | **1.04** | **(1-1.08)** | **52** |
| **Diseases of the blood and blood-forming organs and certain disorders involving the immune mechanism** | **1.05** | **(0.97-1.14)** | **16** |  | **1.00** | **(0.92-1.09)** | **0** |  | **1.04** | **(0.95-1.15)** | **10** |
