# Supplementary material for: Heat-Attributable Deaths between 1992 and 2009 in Seoul, South Korea
Source: PLoS One. 2015 Feb 18;10(2):e0118577. doi: 10.1371/journal.pone.0118577 (PMC4334895; doi:10.1371/journal.pone.0118577)
Supplement: S4 Table — (DOCX) [file pone.0118577.s007.docx]

S4 Table. Sensitivity analysis: Model comparison using different threshold temperature point.

|  | **93% (30.3°C)** | | |  | **95% (31.0°C)** | | |  | **99% (33.4°C)** | | |
| --- | --- | --- | --- | --- | --- | --- | --- | --- | --- | --- | --- |
|  | **RR** | **95% CI** | **AD** |  | **RR** | **95% CI** | **AD** |  | **RR** | **95% CI** | **AD** |
| **All cause** | **1.03** | **(1.03-1.04)** | **2542** |  | **1.04** | **(1.04-1.05)** | **2039** |  | **1.09** | **(1.07-1.11)** | **616** |
| **External causes of morbidity and mortality and injury** | **1.03** | **(1.02-1.05)** | **325** |  | **1.03** | **(1.01-1.05)** | **205** |  | **1.00** | **(0.94-1.05)** | **-3** |
| Transport accidents | 1.01 | (0.98-1.04) | 28 |  | 1.00 | (0.96-1.05) | 9 |  | 0.91 | (0.81-1.02) | -28 |
| **All cardiovascular** | **1.05** | **(1.03-1.06)** | **798** |  | **1.06** | **(1.05-1.07)** | **676** |  | **1.15** | **(1.11-1.19)** | **271** |
| Ischemic heart disease | 1.02 | (1-1.05) | 76 |  | 1.03 | (1-1.07) | 63 |  | 1.12 | (1.03-1.23) | 31 |
| Hypertensive diseases | 1.05 | (1.01-1.09) | 55 |  | 1.06 | (1.01-1.12) | 45 |  | 1.19 | (1.06-1.33) | 23 |
| Heart failure | 1.03 | (0.97-1.09) | 20 |  | 1.05 | (0.98-1.13) | 22 |  | 1.22 | (1.01-1.47) | 12 |
| Myocardial Infarction | 1.03 | (1-1.05) | 67 |  | 1.03 | (1-1.07) | 57 |  | 1.14 | (1.04-1.26) | 29 |
| Stroke, Cerebrovascular diseases | 1.06 | (1.04-1.07) | 559 |  | 1.07 | (1.05-1.09) | 464 |  | 1.17 | (1.12-1.21) | 174 |
| Chronic ischemic heart disease | 1.01 | (0.95-1.08) | 8 |  | 1.01 | (0.94-1.1) | 5 |  | 1.05 | (0.86-1.28) | 2 |
| Sudden Death | 1.06 | (1.01-1.1) | 57 |  | 1.07 | (1.02-1.12) | 50 |  | 1.15 | (1.04-1.26) | 26 |
| **Respiratory System** | **1.03** | **(1-1.05)** | **96** |  | **1.03** | **(1-1.06)** | **77** |  | **1.10** | **(1.01-1.18)** | **31** |
| Asthma | 1.07 | (1.01-1.12) | 46 |  | 1.08 | (1.02-1.15) | 37 |  | 1.17 | (1-1.35) | 12 |
| COPD | 1.01 | (0.97-1.05) | 10 |  | 1.01 | (0.96-1.07) | 7 |  | 1.12 | (0.98-1.28) | 12 |
| Pneumonia | 1.03 | (0.98-1.08) | 31 |  | 1.04 | (0.98-1.1) | 24 |  | 1.09 | (0.93-1.27) | 8 |
| **Endocrine, nutritional and metabolic diseases** | **1.03** | **(1.01-1.06)** | **98** |  | **1.04** | **(1-1.07)** | **68** |  | **1.02** | **(0.94-1.12)** | **7** |
| Diabetes mellitus | 1.03 | (1-1.06) | 80 |  | 1.03 | (1-1.07) | 57 |  | 1.01 | (0.92-1.12) | 4 |
| **Mental and behavioral disorders** | **1.05** | **(1.01-1.09)** | **64** |  | **1.06** | **(1.01-1.11)** | **54** |  | **1.04** | **(0.91-1.18)** | **5** |
| Organic, including symptomatic, mental disorders | 1.04 | (0.99-1.09) | 35 |  | 1.05 | (0.99-1.12) | 30 |  | 1.05 | (0.88-1.24) | 4 |
| PSU | 1.07 | (1-1.14) | 28 |  | 1.08 | (0.99-1.17) | 22 |  | 1.01 | (0.81-1.25) | 0 |
| Schizophrenia | 1.01 | (0.85-1.2) | 1 |  | 1.04 | (0.86-1.27) | 2 |  | 1.11 | (0.72-1.73) | 1 |
| Self-harm | 0.96 | (0.93-0.99) | -118 |  | 0.94 | (0.9-0.98) | -101 |  | 0.91 | (0.8-1.03) | -19 |
| **Diseases of the digestive system** | **1.01** | **(0.99-1.04)** | **59** |  | **1.02** | **(0.99-1.05)** | **44** |  | **1.03** | **(0.95-1.11)** | **10** |
| **Diseases of the nervous system** | **1.10** | **(1.06-1.15)** | **109** |  | **1.14** | **(1.08-1.2)** | **93** |  | **1.29** | **(1.15-1.46)** | **30** |
| **Diseases of the genitourinary system** | **1.06** | **(1.02-1.11)** | **74** |  | **1.08** | **(1.03-1.13)** | **59** |  | **1.23** | **(1.09-1.39)** | **25** |
| **Diseases of the blood and blood-forming organs and certain disorders involving the immune mechanism** | **1.03** | **(0.92-1.14)** | **5** |  | **1.05** | **(0.92-1.19)** | **6** |  | **1.12** | **(0.81-1.53)** | **2** |
